# P-712. Increased risk of wheezing episodes in the first year after RSV associated hospitalization in children ≤2 years

**DOI:** 10.1093/ofid/ofaf695.924

**Published:** 2026-01-11

**Authors:** Joanne G Wildenbeest, Louis J Bont, Rebecca Stellato, Johannes Liese, Egbert Herting, Renato Cutrera, Christina Calvo, Jacques Brouard, Chiara Azzari, Federico Martinon-Torres, Simon Drysdale, Atul Gupta, Ralph Epaud, Madelyn Ruggieri, Yoonyoung Choi

**Affiliations:** UMC Utrecht, Utrecht, Utrecht, Netherlands; University Medical Centre Utrecht, Utrecht, Utrecht, Netherlands; Utrecht University, Utrecht, Utrecht, Netherlands; UKW Würzburg, Würzburg, Bayern, Germany; University of Lübeck, D 23562 Lübeck, Schleswig-Holstein, Germany; Bambino Gesù Children's Hospital, Rome, Lazio, Italy; Hospital Universitario La Paz. Universidad Autónoma de Madrid, Madrid, Madrid, Spain; CHU Caen, Caen, Basse-Normandie, France; University of Florence and Anna Meyer Children’s Hospital, Florence, Toscana, Italy; Hospital Clínico Universitario de Santiago, Santiago de Compostela, Spain, Santiago de Compostela, Galicia, Spain; University of Oxford, Oxford, England, United Kingdom; King's college Hospital, London, England, United Kingdom; Hopital Intercommunal de Créteil, Creteil, Creteil, Ile-de-France, France; Merck, Philadelphia, Pennsylvania; Merck, Philadelphia, Pennsylvania

## Abstract

**Background:**

Respiratory syncytial virus (RSV) is a leading cause of hospitalizations among young children globally. However, the impact of RSV-associated hospitalization on subsequent wheezing episodes remains understudied in Europe. We assessed the incidence rate ratio (IRR) of wheezing in the first year after hospitalization in children hospitalized with RSV compared to those hospitalized without acute respiratory infections (ARI).

**Methods:**

We conducted a prospective matched cohort study in hospitalized children aged ≤2 years across ten hospitals in Germany, Spain, Italy, France, and England from October 2020 to May 2023. We compared the incidence rates of caregiver-reported wheezing, medically attended wheezing, and wheezing requiring medication within 12 months post-discharge in children with laboratory-confirmed RSV to matched children admitted for non-ARI. Caregivers reported wheezing episodes through bi-monthly online questionnaires. Potential confounders were adjusted using propensity scores as covariates in generalized estimating equations.

**Results:**

A total of 300 hospitalized children with RSV and 140 with non-ARI were included, with an average 89% completion rate for the bi-monthly questionnaires. Two-thirds (66%) of children admitted with RSV experienced caregiver-reported wheezing episodes, compared to only 26% of non-ARI children; with an IRR of 3.58 (95% CI 2.87–4.46). The adjusted IRRs for caregiver-reported wheezing episodes, medically attended wheezing episodes, and use of respiratory medication were 2.51 (95% CI 1.46–4.33), 2.29 (95% CI 1.50–3.52), and 2.91 (95% CI 1.90–4.47), respectively. These associations remained consistent after imputation for missing data.

**Conclusion:**

Hospitalization due to RSV in children aged ≤2 years is associated with a significantly increased risk of wheezing in the year following discharge, underscoring the long-term respiratory burden of RSV infection in early childhood.

**Disclosures:**

Joanne G. Wildenbeest, MD, PhD, Merck & Co., Inc.: Grant/Research Support|Merck & Co., Inc.: Presentation at sponsored symposium|Sanofi: Grant/Research Support|Sanofi: participation in advisory boards, speaker at a Sanofi sponsored symposium Louis J Bont, M.D., Merck: Advisor/Consultant|Merck: Grant/Research Support Rebecca Stellato, n/a, Merck & Co., Inc.: Grant/Research Support Johannes Liese, MD MSc, Sanofi: Grant/Research Support Egbert Herting, PhD, Chiesi: Grant/Research Support Renato Cutrera, MD, Merck & Co., Inc.: Grant/Research Support Christina Calvo, MD, PhD, Merck & Co., Inc.: Grant/Research Support Chiara Azzari, Merck & Co., Inc.: Grant/Research Support Federico Martinon-Torres, MD, PhD, Assoc. Prof, Astra Zeneca: Advisor/Consultant|Astra Zeneca: Board Member|Astra Zeneca: Grant/Research Support|Astra Zeneca: Honoraria|Astra Zeneca: trial fees as PI, paid to may institution|GSK: Advisor/Consultant|GSK: Board Member|GSK: Grant/Research Support|GSK: Honoraria|GSK: trial fees as PI, paid to may institution|Merck & Co., Inc.: Advisor/Consultant|Merck & Co., Inc.: Grant/Research Support|Merck & Co., Inc.: Honoraria|Merck & Co., Inc.: trial fees as PI, paid to may institution|Moderna: Advisor/Consultant|Moderna: Grant/Research Support|Moderna: trial fees as PI, paid to may institution|Pfizer: Advisor/Consultant|Pfizer: Board Member|Pfizer: Grant/Research Support|Pfizer: Honoraria|Pfizer: trial fees as PI, paid to may institution|Sanofi Pasteur: Advisor/Consultant|Sanofi Pasteur: Board Member|Sanofi Pasteur: Grant/Research Support|Sanofi Pasteur: Honoraria|Sanofi Pasteur: trial fees as PI, paid to may institution|Seqirus: Advisor/Consultant|Seqirus: Grant/Research Support|Seqirus: trial fees as PI, paid to may institution Simon Drysdale, FRCPCH, MSD: Grant/Research Support Ralph Epaud, MD, ASTRA: Advisor/Consultant|ASTRA: Board Member|GSK: Advisor/Consultant|SANOFI: Board Member Madelyn Ruggieri, MS, Merck: employee of Merck Yoonyoung Choi, PhD, MS, RPh, Merck & Co., Inc.: Grant/Research Support|Merck & Co., Inc.: Employment|Merck & Co., Inc.: Stocks/Bonds (Private Company)

